# Loss of *GABARAPL1* confers ferroptosis resistance to cancer stem‐like cells in hepatocellular carcinoma

**DOI:** 10.1002/1878-0261.13305

**Published:** 2022-09-05

**Authors:** Xiaojing Du, Zhuoran Qi, Jinzhi Xu, Mengzhou Guo, Xingxing Zhang, Zhijie Yu, Xin Cao, Jinglin Xia

**Affiliations:** ^1^ Department of Gastroenterology, Minhang Hospital Fudan University Shanghai China; ^2^ Key Laboratory of Diagnosis and Treatment of Severe Hepato‐Pancreatic Diseases of Zhejiang Province The First Affiliated Hospital of Wenzhou Medical University China; ^3^ Liver Cancer Institute, Zhongshan Hospital Fudan University Shanghai China; ^4^ Department of Gastroenterology Anhui University of Science and Technology Affiliated Fengxian Hospital, Shanghai Fengxian District Central Hospital China; ^5^ Wenzhou Key Laboratory of Hematology The First Affiliated Hospital of Wenzhou Medical University China; ^6^ Institute of Clinical Science, Zhongshan Hospital Fudan University Shanghai China

**Keywords:** cancer stem‐like cell, ferroptosis, *GABARAPL1*, hepatocellular carcinoma, sorafenib

## Abstract

Cancer stem‐like cells (CSLC) are considered a major contributor to the development and progression of hepatocellular carcinoma (HCC). Previous studies indicated that CSLC are characterized by resistance to ferroptosis, a type of lipid peroxidation‐dependent cell death. Here, we identified a set of ferroptosis‐related stemness genes (FRSG) and found that these genes may be involved in immune infiltration in HCC. A four‐FRSG (*CDKN2A*, *GABARAPL1*, *HRAS*, *RPL8*) risk model with prognostic prediction was constructed by a Cox analysis in HCC. Among these four genes, *GABARAPL1* was downregulated in HCC tumor‐repopulating cells (TRC; a type of CSLC). Its downregulation decreased the sensitivity of HCC TRC to erastin‐ or sorafenib‐triggered ferroptosis. Together, we uncovered a molecular mechanism via which CSLC could achieve tolerance to ferroptosis. Further studies may provide potential therapeutic strategies targeting CSLC in HCC.

## Graphical abstract

Ferroptosis‐related stemness genes (FRSG) were found to be significantly associated with the immunity and prognosis of hepatocellular carcinoma (HCC). An FRSG‐based risk score effectively predicted HCC patient prognosis, and loss of *GABARAPL1* conferred ferroptosis resistance of HCC cancer stem‐like cells, while overexpressing it may recover sensitivity.

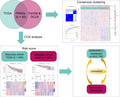

**Abbreviations**
ATG8: autophagy‐related 8
AUC: under the curve
BP: biological processes
CSLC: cancer stem‐like cells
DDS: disease‐free survival
DEG: differentially expressed genes
DMEM: Dulbecco's modified Eagle medium
FBS: fetal bovine serum
FRG: ferroptosis‐related genes
FRSG: ferroptosis‐related stemness genes
GO: Gene Ontology
GSH: glutathione
HBV: hepatitis B virus
HCC: hepatocellular carcinoma
HPA: the Human Protein Atlas
ICGC: International Cancer Genome Consortium
ICI: immune checkpoint inhibitors
K–M: Kaplan–Meier
MEM: Minimum Essential Medium
mRNAsi: mRNA expression‐based stemness index
OCLR: one‐class logistic regression
OS: overall survival
PFS: progression free survival
RCD: regulated cell death
RFS: recurrence‐free survival
ROC: receiver operating characteristic
RS: risk score
system x_c_^−^
: cystine/glutamate transporter
TCGA: The Cancer Genome Atlas
TIDE: tumor immune dysfunction and exclusion
TMB: tumor mutational burden
TRC: tumor‐repopulating cells

## 1 Introduction

Hepatocellular carcinoma (HCC) is a major type of primary liver cancer. Although its treatments have led to great improvements, patients with HCC still suffer a poor outcome, being mainly due to the limited efficacy of treatment as well as primary or acquired resistance [1]. Cancer stem‐like cells (CSLC) attract a growing body of attentions for their features (e.g. self‐renewal and differentiation) that could regenerate all properties of a tumor [2]. Convincing evidence indicates the driving role of CSLC in HCC, greatly contributing to the tumor recurrence and therapy resistance [2]. Therefore, targeting CSLC was considered a potential therapeutic strategy for HCC.

Ferroptosis is a novel iron‐dependent regulated cell death (RCD) caused by unlimited lipid peroxidation and subsequent membrane destruction [3]. It is regulated via two pathways – an extrinsic pathway mediated by the suppression of cell membrane transporters (e.g. cystine/glutamate transporter, also known as system xc^−^) or by the activation of iron transporter, and an intrinsic pathway triggered by the inhibition of antioxidant cascades (e.g. GPX4) [3]. Of note, most investigations agree that there is a higher iron level in CSLC than in differentiated tumor cells [4]. Excess iron can induce ferroptosis via a Fenton reaction [5], indicating that triggering ferroptosis may be a selective and potential strategy for killing CSLC. However, a high level of iron conferred robust sphere‐forming capacity and stemness rather than ferroptosis to CSLC in some cancer types [4], prompting the suggestion CSLC may evolve countermeasures against ferroptosis. For instance, pluripotency factor SOX2 transcriptionally upregulates SLC7A11, a key component of the system xc^−^, and thus accelerates cysteine uptake and glutathione (GSH) synthesis, and confers ferroptosis resistance to CSLC in lung cancer [6]. Consequently, it is of great importance to understand the mechanism underlying CSLC resistance to ferroptosis, contributing to the strategy for killing CSLC.

Bioinformatics has attracted growing attention due to its potent function in mining the molecular mechanism in cancers. In the paper, a one‐class logistic regression (OCLR) machine‐learning algorithm was used to identify ferroptosis‐related stemness genes (FRSG) in HCC [7]. Consensus clustering was first employed to classify HCC samples from The Cancer Genome Atlas (TCGA) database and evaluated its correlation with immune infiltration and tumor mutational burden (TMB). Cox analysis was then applied to construct a four‐gene risk score (RS) model for predicting patient survival. Most importantly, we revealed that one of the hub genes, *GABARAPL*, was downregulated in tumor‐repopulating cells (TRC, a kind of CSLC) [8], which was associated with TRC resistance to ferroptosis inducers (erastin and sorafenib).

## 2 Methods and materials

### 2.1 Bioinformatics analysis

HCC RNA‐seq data (TCGA‐LIHC) were downloaded from TCGA (normal, *n* = 47; tumor, *n* = 345; https://portal.gdc.cancer.gov/) and the ICGC database (International Cancer Genome Consortium; tumor, *n* = 231; https://dcc.icgc.org/projects/LIRI‐JP). The clinical information of these HCC patients is displayed in Table 1. The RNA‐seq data of other 32 types of cancers were also downloaded from TCGA database. The OCLR algorithm was applied to calculate the mRNA expression‐based stemness index (mRNAsi) of each sample from TCGA [7]. Ferroptosis‐related genes (FRG, *n* = 248) were obtained from the FerrDb database (http://www.zhounan.org/ferrdb/legacy/index.html#) [9]. The package ‘limma’ of R (version 4.1.0) was employed to identify the differential expressed genes (DEG) between normal and HCC tissues based on TCGA [10]. Gene Ontology (GO) analysis was conducted by using the ‘clusterProfiler’ package [11].

**Table 1:** Clinical features of tumor patients in TCGA and ICGC datasets.

Characteristics	TCGA (*n* = 345)	ICGC (*n* = 231)
Age		
< 65	205	82
≥ 65	139	149
Sex		
Male	235	170
Female	110	61
Stage		
I	162	36
II	78	105
III	80	71
IV	3	19
Grade		
G1	53	
G2	162	
G3	113	
G4	12	
NA	5	
Body mass index		
< 18.5	19	
18.5–24.9	144	
≥ 25	154	
NA	28	
Fibrosis Ishak score		
0 – no fibrosis	72	
1,2 – portal fibrosis	30	
3,4 – fibrous septa	24	
5 – nodular formation and incomplete cirrhosis	8	
6 – established cirrhosis	67	
NA	141	

Consensus clustering achieved by the ‘ConsensusClusterPlus’ package was employed to classify HCC patients [12]. Kaplan–Meier (K–M) curve was plotted using the ‘survival’ package (https://cran.r‐project.org/package=survival) to achieve the survival analysis. The tumor microenvironment was evaluated by ESTIMATE, MCPcounter and ssGSEA algorithms [13, 14, 15]. The tumor immune dysfunction and exclusion (TIDE) algorithm was used to predict patients' responses to immune checkpoint inhibitors (ICI) [16]. The mutation information was obtained from TCGA and analyzed using the ‘maftools’ package [17].

According to the discovery cohort TCGA, univariate Cox analysis was employed to confirm the candidate genes with prognostic signature (*P* < 0.05 as the threshold). Multivariate Cox analysis (*P* < 0.05 as the cutoff value) was thenapplied to construct a risk model as followed: RS = ∑Coef_mRNAs_ × Exp_mRNAs_. The model was further estimated by the K–M curve and receiver operating characteristic (ROC) curve in both the discovery cohort (TCGA) and the validation cohort (ICGC). Subsequently, univariate Cox analysis proceeded to estimate the clinical characteristics and RS, with *P* < 0.2 as the cutoff value. Further multivariate Cox analysis identified the factors for a constructing nomogram, with *P* < 0.05 as the threshold. Considering the importance of stage, the nomogram was established by variables including RS, sex and stage, using the ‘rms’ package (https://cran.r‐project.org/package=rms). Moreover, the protein expression of *GABARAPL1* was investigated using the Human Protein Atlas (HPA) database (https://www.proteinatlas.org/) [18]. Finally, survival analysis of *GABARAPL1* in HCC was conducted by the Kaplan–Meier Plotter online tool [19].

### 2.2 Cell lines and reagents

All cell lines were from the Liver Cancer Institute, Zhongshan Hospital, Fudan University (Shanghai, China). Human liver cancer cell lines SNU449, SK‐Hep1, MHCC97H, SMMC7721, LM3, Huh7 and PLC/PRF/5, and normal human liver cell line L02 were maintained in Dulbecco's modified Eagle medium (DMEM; GNM12800‐2, GENOM, Zhejiang, China) with 10% FBS (Sigma, St. Louis, MO, USA) and 1% penicillin–streptomycin (GNM‐15140, GENOM). Human liver cancer cell line Hep3B was cultured in Minimum Essential Medium (MEM; GNM41500‐2, GENOM) with 10% FBS and 1% penicillin–streptomycin. Cells were kept at 37 °C in 5% CO2 in a humidified ThermoForma incubator (Thermo Fisher Scientific, Waltham, MA, USA).

Erastin (HY‐15763), RSL3 (HY‐100218A) and sorafenib (HY‐10201) were purchased from MedChemExpress (Monmouth Junction, NJ, USA). *GABARAPL1* (#26632) antibody (Ab) was from Cell Signaling Technology (Beverly, MA, USA), and GAPDH Ab (AF0006) was sourced from Beyotime Biotechnology (Nantong, China). Rabbit (A0208) and mouse (A0216) horseradish peroxidase‐conjugated secondary Ab were from Beyotime Biotechnology. Salmon fibrinogen (SEA‐133) and thrombin (SEA‐135) were purchased from Sea Run Holdings Inc. (Freeport, ME, USA).

### 2.3 3D culture of TRC

Soft 3D fibrin gel was established to select TRC. Previous study indicated that fibrin gel of 90 Pa was the optimal gel for cancer cell spheroid formation [20]. Hence, to screen HCC TRC, cells cultured in 2D rigid plates were trypsinized and resuspended with complete medium, followed by mixing with an isochoric salmon fibrinogen (2 mg·mL^−1^) diluted with T7 buffer (50 mm Tris–HCl, 150 mm NaCl, pH 7.4). Then, 100 U·mL^−1^ thrombin was added to culture plate, and mixed with the cell suspension (Volume, thrombin/cell suspension 1 : 50). After incubation for 30 min in 37 °C humidified incubator, the 3D fibrin gel was finished and then complete medium was added to each well. Finally, the cells were cultured in humidified incubator and used for further experiments.

### 2.4 Colony formation assay

Colony formation assay was performed to determine the proliferation ability of 2D cultured HCC cells. In short, cells (1–3 × 10^4^ cells per well) were incubated in a 6‐well plate and cultured for 7–14 days. Colonies were then stained with crystal violet solution (V5265, Sigma). After recording, the crystal violet in each well was eluted by 30% acetic acid (1 mL) and 100 μL eluent was added to a 96‐well plate and its absorbance was measured at 600 nm using FlexStation3 multi‐mode (Molecular Devices Corporation, Sunnyvale, CA, USA).

### 2.5 Western blotting assay

Total proteins were obtained from HCC cells in 2D/3D cultures applying RIPA lysis buffer (WB0101, WellBio Technology Co., Ltd, Shanghai, China), and were separated on 12% SDS/PAGE and then transferred to polyvinylidene difluoride (PVDF; ISEQ00010, Merck Millipore Ltd., Darmstadt, Germany) membranes. After blocking by western blocking buffer (WB6014, WellBio Technology Co., Ltd) for 30 min, the membranes were probed using the primary Ab at 4 °C overnight and then incubated with corresponding secondary Ab for 1 h. Finally, the bands were detected by Chemistar™ high‐sig ECL western blotting substrate (180–5001, Tanon, Shanghai, China) and visualized by Odyssey Imaging System (LiCor Biosciences, Lincoln, NE, USA).

### 2.6 Quantitative reverse transcription polymerase chain reaction (qRT‐PCR)

Total RNA were extracted from HCC cells in 2D/3D cultures using TRIzol reagent (15596026, Invitrogen, Carlsbad, CA, USA). Complementary DNA was obtained using PrimeScript RT Master Mix (RR036A, TaKaRa, Dalian, China). Then qRT‐PCR was performed using SYBR Green kit (11202ES08, Yeasen, Shanghai, China) on an ABI Prism 7500 sequence detection system (Applied Biosystems, Foster City, CA, USA). The conditions were set as follows: initial denaturation at 95 °C for 5 min, 40 cycles of 95 °C for 10 s and 60 °C for 30 s. The $2^{-\Delta \Delta C_{T}}$ method was used to calculate the gene expression change, with GAPDH as the internal normalization. The primes were:

*GAPDH* forward primer 5′‐GGAAGCTTGTCATCAATGGAAATC‐3′;
*GAPDH* reverse primer 5′‐TGATGACCCTTTTGGCTCCC‐3′;
*GABARAPL1* forward primer 5′‐AGGGTCCCCGTGATTGTAGA‐3′;
*GABARAPL1* reverse primer 5′‐AGAACTGGCCAACAGTAAGG‐3′.

### 2.7 Lipid peroxidation and GSH assay

According to the manufacturer's instructions, lipid peroxidation was detected using MDA assay kit (S0131S, Beyotime) and BODIPY 581/591 C11 (D3861, Invitrogen), and the GSH level was measured by applying a Glutathione Assay Kit (Beyotime, S0053). The level of MDA and GSH was normalized to the corresponding protein level.

### 2.8 RNA interference and lentivirus‐mediated transfection

To silence the expression of *GABARAPL1*, cells were transfected with siRNA by applying riboFECT™ CP (C10511‐05, RIBOBIO, Guangdong, China). The siRNA sequence targeting *GABARAPL1* was 5′‐GGACCAUCCCUUUGAGUAUUU‐3′, and control siRNA was 5′‐UAAGGCUAUGAAGAGAUACUU‐3′.

To overexpress *GABARAPL1*, lentivirus was used to transfect the plasmid into HCC cells. Plasmid for overexpressing *GABARAPL1* was purchased from Genomeditech Co., Ltd. (Shanghai, China).

### 2.9 Statistical analysis

r software (version 4.1.0, R Core Team, R Foundation for Statistical Computing, Vienna, Austria) and graphpad prism 8 (San Diego, CA, USA) were employed for plotting and statistical analysis. The RS model was established using univariate and multivariate Cox analysis. The *t*‐test and Mann–Whitney test were applied for normally and non‐normally distributed data, respectively. The overall survival (OS) represented the time from diagnosis to the last follow‐up or death. As for the cellular experiments, *t*‐test was used to compare the difference between two groups. The data were displayed as mean ± SD of three independent experiments. *P* < 0.05 was considered statistically significant unless otherwise noted.

## 3 Results

### 3.1 Identification of DEG related to both ferroptosis and stemness

**Fig. 1:**
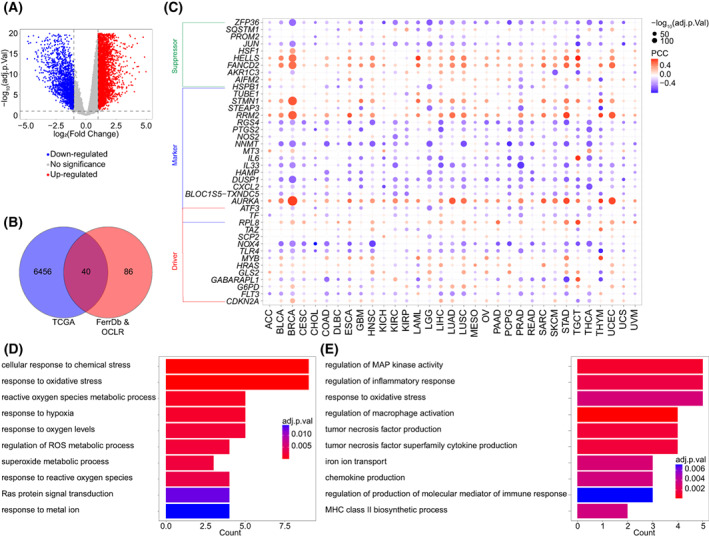
Identification of FRSG in HCC. (A) DEG in TCGA dataset (normal, *n* = 47; tumor, *n* = 345). (B) The stemness‐related FRG were determined by Pearson correlation analysis in TCGA dataset (*n* = 345). Venn diagram shows the overlapped genes between DEG in TCGA and stemness‐related FRG screened by OCLR algorithm. (C) Pearson correlation analysis was used to calculate the association between FRSG and stemness in various cancer types, including ACC (*n* = 79), BLCA (*n* = 411), BRCA (*n* = 1118), CESC (*n* = 306), CHOL (*n* = 32), COAD (*n* = 471), DLBC (*n* = 48), ESCA (*n* = 162), GBM (*n* = 168), HNSC (*n* = 502), KICH (*n* = 65), KIRC (*n* = 535), KIRP (*n* = 289), LAML (*n* = 151), LGG (*n* = 529), LIHC (*n* = 345), LUAD (*n* = 527), LUSC (*n* = 501), MESO (*n* = 86), OV (*n* = 379), PAAD (*n* = 178), PCPG (*n* = 183), PRAD (*n* = 500), READ (*n* = 167), SARC (*n* = 263), SKCM (*n* = 471), STAD (*n* = 375), TGCT (*n* = 156), THCA (*n* = 512), THYM (*n* = 119), UCEC (*n* = 548), UCS (*n* = 56), UVM (*n* = 80). (D, E) GO analysis revealed the enriched BP of upregulated (D) and downregulated (E) FRSG. FRSG, ferroptosis‐related stemness genes; HCC, hepatocellular carcinoma; DEG, differential expressed genes; TCGA, the cancer genome atlas; OCLR, one‐class logistic regression; FRG, ferroptosis‐related genes; GO, gene ontology; BP, biological processes.

According to TCGA dataset, 6496 DEG were identified between normal and HCC tissues, consisting of 4666 upregulated and 1830 downregulated DEG (Fig. 1A). We next obtained 248 FRG from the FerrDb database and evaluated their correlation with tumor stemness via OCLR algorithm and Pearson correlation analysis, which identified 126 FRSG (Fig. 1B). The overlapped genes (*n* = 40) between the two sets were considered as the FRSG in HCC, including 19 upregulated and 21 downregulated FRSG (Fig. 1B). We also showed the potential correlation between these genes and stemness in a broad range of cancer types (Fig. 1C). GO analysis indicated that the top three enriched biological processes (BP) of upregulated FRSG were cellular response to chemical stress, response to oxidative stress and reactive oxygen species metabolic process (Fig. 1D). The downregulated FRSG were mainly concentrated on regulation of MAP kinase activity, regulation of inflammatory response and response to oxidative stress (Fig. 1E).

### 3.2 C2 type HCC possess longer OS and higher immune infiltration, but lower TMB

**Fig. 2:**
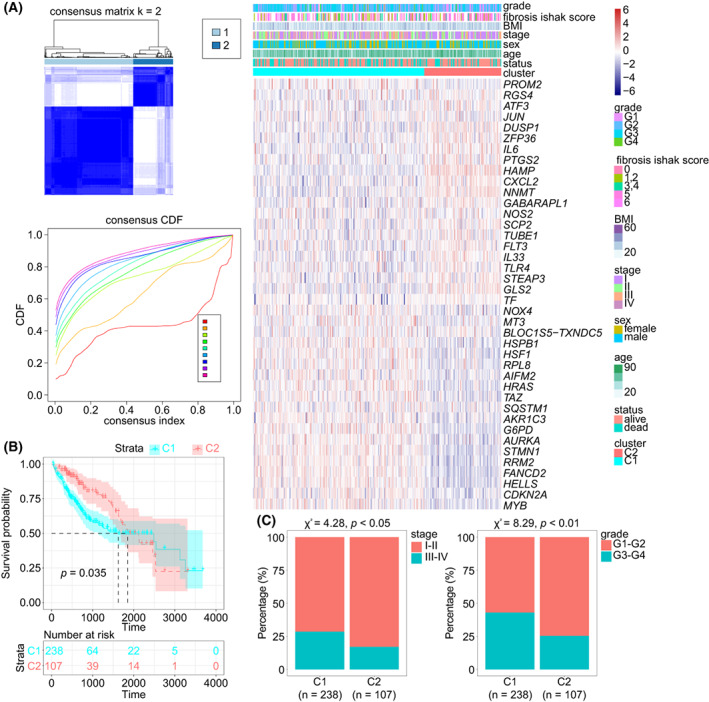
Consensus clustering based on FRSG in TCGA dataset. (A) HCC samples were classified into C1 and C2 type by consensus clustering (*n* = 345). (B) Survival analysis of the C1 (*n* = 238) and C2 (*n* = 107) type of HCC (log rank test). (C) Comparison of clinical features in the C1 (*n* = 238) and C2 (*n* = 107) type of HCC (Chi‐square test). FRSG, ferroptosis‐related stemness genes; HCC, hepatocellular carcinoma; TCGA, The Cancer Genome Atlas.

**Fig. 3:**
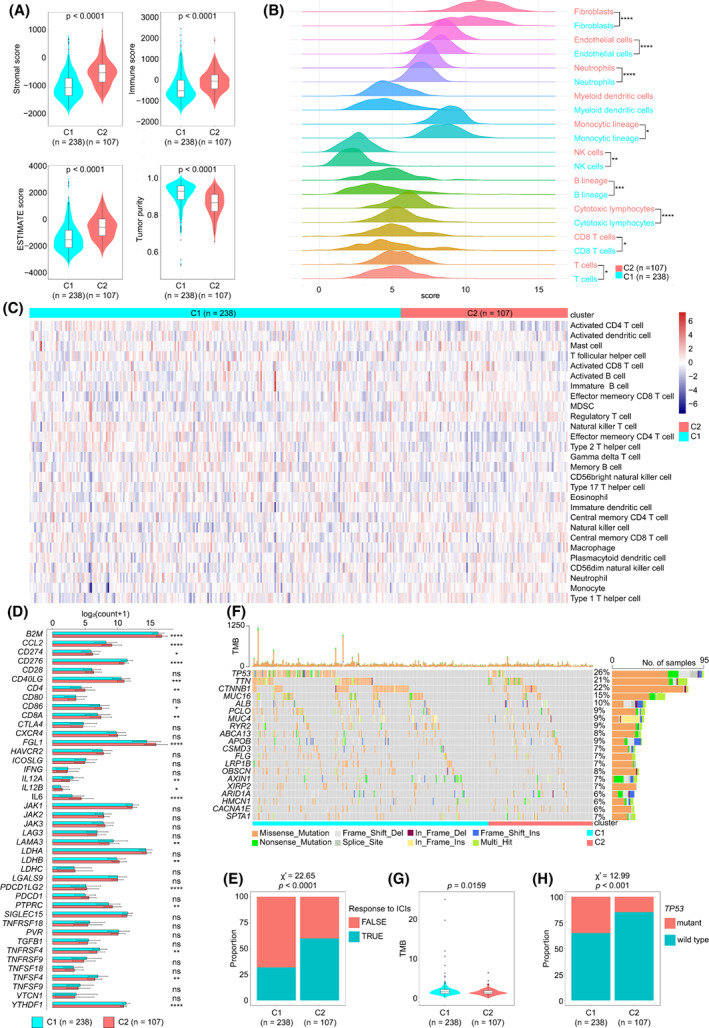
Microenvironment and mutation estimation of C1 and C2 type HCC. (A–C) The immune microenvironment of HCC was determined by ESTIMATE (A), MCPcounter (B) and ssGSEA (C) algorithms. (D) The expression of immune checkpoints in HCC. The error bars indicate SD. (E) TIDE algorithm showed differential ICI sensitivity in the two clusters (Chi‐square test). (F) Waterfall plot shows the mutation frequency in C1 and C2 type HCC. (G) TMB analysis. (H) The frequency of TP53 mutation in HCC (Chi‐square test). All of these analyses were conducted in TCGA‐LIHC cohort (*n* = 345). When comparing two groups, the *t*‐test and Mann–Whitney test were applied for normally and non‐normally distributed data, respectively. HCC, hepatocellular carcinoma; ICI, immune checkpoint inhibitors; TIDE, tumor immune dysfunction and exclusion. *P < 0.05, **P < 0.01, ***P < 0.001, ****P < 0.0001.

Consensus clustering was employed to classify HCC samples into two clusters, C1 and C2 (Fig. 2A). The heatmap displayed the expression of the FRSG in the two clusters. K–M curve indicated that C2 type HCC had a longer OS than C1 type HCC (mOS, 1852 *vs*. 1622 days, *P* = 0.035; Fig. 2B). More advanced (28.5% *vs*. 17.0%, *P* < 0.05) and high grade (43.0% *vs*. 25.3%, *P* < 0.01) tumors were observed in C1 than in C2 type HCC (Fig. 2C). We then estimated the immune infiltration and found that C2 type HCC had a higher stromal score (*P* < 0.0001), immune score (*P* < 0.0001) and ESTIMATE score (*P* < 0.0001), but lower tumor purity (*P* < 0.0001) compared with C1 type HCC (Fig. 3A). MCPcounter and ssGSEA algorithms were employed to investigate the infiltrated cells and demonstrated that C2 type HCC had higher infiltration than C1 type HCC (Fig. 3B,C). Further analysis revealed that the majority of immune checkpoints had a higher expression in C2 than in C1 type HCC, including *CD274*, *PDCD1LG2* and *CD40LG* (Fig. 3D). TIDE algorithm was next applied to predict patients' responses to ICI. The data showed that C2 type HCC was more reactive to ICI than was C1 type HCC (59.7% *vs*. 31.9%, *P* < 0.0001; Fig. 3E). We also evaluated the somatic mutations between the two clusters. According to the waterfall plot, more mutations occurred in C1 than in C2 type HCC (Fig. 3F). The TMB was calculated and revealed that C1 type HCC had higher TMB than C2 type HCC (*P* = 0.0159; Fig. 3G). The mutation rate of *TP53*, the most frequent mutation in HCC, was also higher in C1 than in C2 type HCC (35.1% *vs*. 14.9%, *P* < 0.001; Fig. 3H). Taken together, C2 type HCC had better prognosis, higher immune infiltration ad a higher response to ICI, but a lower TMB compared with C1 type HCC.

### 3.3 Construction and validation of an RS model with prognostic signature

**Fig. 4:**
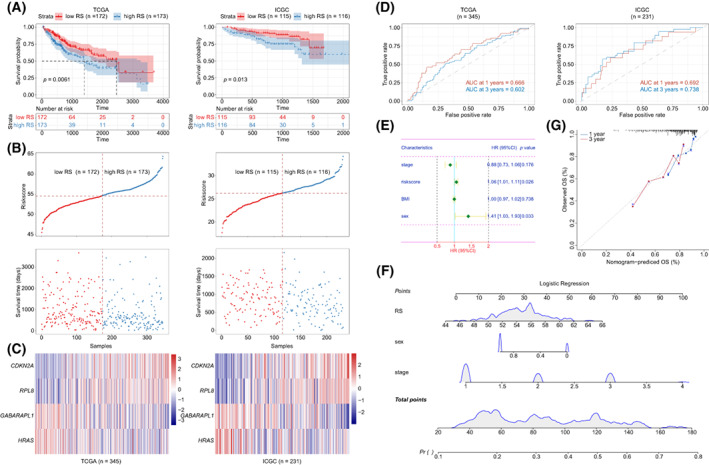
Construction of an RS model and nomogram. (A) K–M curve of RS in TCGA (*n* = 345) and ICGC (*n* = 231) datasets (log rank test). (B) RS for each sample in TCGA (*n* = 345) and ICGC (*n* = 231) datasets were displayed in scatter diagram. (C) The expression of the hub genes in TCGA (*n* = 345) and ICGC (*n* = 231) datasets. (D) ROC curve of RS in TCGA (*n* = 345) and ICGC (*n* = 231) datasets. (E) Forest diagram showed the multivariable Cox analysis of clinical features and RS in TCGA (*n* = 345) dataset. The error bars indicate 95% confidence interval (CI). (F, G) A nomogram was established in TCGA (*n* = 345) dataset based on RS, sex and stage (F). The efficacy of nomogram was estimated by calibration curves in TCGA (*n* = 345) dataset (G). ICGC, International Cancer Genome Consortium; AUC, under the curve; K–M, Kaplan–Meier; OS, overall survival; ROC, receiver operating characteristic; RS, risk score; TCGA, The Cancer Genome Atlas.

To construct a model for predicting HCC OS, univariate Cox analysis was used to identify the candidate genes with prognostic signature in the discovery cohort TCGA dataset. Taking *P* < 0.05 as threshold, 20 candidate genes were screened and further analyzed using multivariate Cox analysis (Table 2). A risk model was established as follows: RS = 1.14 × Exp *CDKN2A* + 1.19 × Exp *GABARAPL1* + 0.76 × Exp *HRAS* + 1.48 × Exp *RPL8*. HCC patients were divided into two groups, a low RS group and a high RS group, based on the median value. The K–M curve indicated that the high RS group possessed significantly shorter OS than the low RS group (1397 *vs*. 2456 days, *P* < 0.0061; Fig. 4A). Increasing tumor‐related death and decreasing patient survival occurred with increasing RS, and the expression of related FRSG was displayed in a heatmap (Fig. 4B,C). The area under the curve (AUC) values of RS were 0.666 and 0.602 for 1‐year survival and 3‐year survival, respectively (Fig. 4D). The predictive efficiency of RS was also verified in the ICGC dataset (Fig. 4A–D).

**Table 2:** Univariable and multivariable Cox analysis of the candidate genes.

Genes	Univariable Cox		Multivariable Cox	
	HR [95% CI]	*P*‐value	HR [95% CI]	*P*‐value
*AIFM2*	1.40 [1.12–1.73]	0.002473	1.01 [0.82–1.25]	0.923
*AKR1C3*	1.33 [1.13–1.57]	0.000685	0.96 [0.79–1.17]	0.712
*AURKA*	1.29 [1.13–1.48]	0.000264	1.08 [0.91–1.28]	0.382
*BLOC1S5‐TXNDC5*	1.14 [1.02–1.27]	0.022668	0.90 [0.79–1.02]	0.104
***CDKN2A***	**1.18 [1.07–1.30]**	**0.001195**	**1.14 [1.04–1.26]**	**0.007**
*FANCD2*	1.31 [1.15–1.50]	4.72E‐05	1.13 [0.87–1.46]	0.355
*FLT3*	0.90 [0.81–1.00]	0.046235	1.00 [0.91–1.09]	0.939
*G6PD*	1.41 [1.27–1.57]	1.22E‐10	0.99 [0.84–1.16]	0.87
*GABARAPL1*	**0.83 [0.72–0.96]**	**0.012061**	**1.19 [1.02–1.39]**	**0.027**
*HELLS*	1.22 [1.09–1.36]	0.000636	1.07 [0.89–1.29]	0.456
***HRAS***	**1.48 [1.22–1.78]**	**5.35E‐05**	**0.76 [0.58–0.98]**	**0.035**
*HSF1*	1.47 [1.19–1.82]	0.000321	0.75 [0.52–1.08]	0.126
*HSPB1*	1.23 [1.05–1.43]	0.009336	1.09 [0.90–1.31]	0.374
*MT3*	1.23 [1.05–1.20]	0.000715	1.04 [0.97–1.11]	0.268
*MYB*	1.25 [1.12–1.39]	7.60E‐05	1.02 [0.91–1.15]	0.735
***RPL8***	**1.27 [1.08–1.49]**	**0.002956**	**1.48 [1.13–1.94]**	**0.004**
*RRM2*	1.32 [1.17–1.50]	1.17E‐05	0.88 [0.72–1.09]	0.246
*SQSTM1*	1.41 [1.20–1.65]	2.14E‐05	1.03 [0.87–1.22]	0.735
*STMN1*	1.46 [1.26–1.68]	3.05E‐07	0.85 [0.68–1.07]	0.162
*TAZ*	1.47 [1.12–1.93]	0.005725458	1.00 [0.75–1.32]	0.978

The bold values indicate the significant factors calculated by both univariable and multivariable cox analysis.

Next, univariate and multivariate Cox analysis indicated that RS and sex were independent prognostic factors for HCC OS (Table 3, Fig. 4E). Given the importance of tumor stage, they were employed together to construct a nomogram (Fig. 4F). Compared with the ideal model in TCGA dataset, calibration plots suggested that 1‐year and 3‐year OS rates were well predicted in HCC patients by this nomogram (Fig. 4G).

**Table 3:** Univariable and multivariable Cox analysis of the clinical features and risk score.

Clinical features	Univariable Cox		Multivariable Cox	
	HR [95% CI]	*P*‐value	HR [95% CI]	*P*‐value
Age	1.01 [0.99–1.02]	0.248759		
Body mass index	0.97 [0.93–1.00]	0.065496	1.00 [0.97–1.02]	0.738
Grade	1.09 [0.86–1.39]	0.488169		
Fibrosis Ishak score	0.90 [0.77–1.06]	0.205068		
**Risk score**	**1.11 [1.05–1.17]**	**0.000463**	**1.06 [1.01–1.11]**	**0.026**
**Sex**	**0.78 [0.54–1.12]**	**0.171482**	**1.41 [1.03–1.93]**	**0.033**
Stage	1.8 [1.46–2.23]	4.25E‐08	0.88 [0.73–1.06]	0.176

The bold values indicate the significant factors calculated by both univariable and multivariable cox analysis.

### 3.4 Low *GABARAPL1* is associated with poor prognosis of HCC

**Fig. 5:**
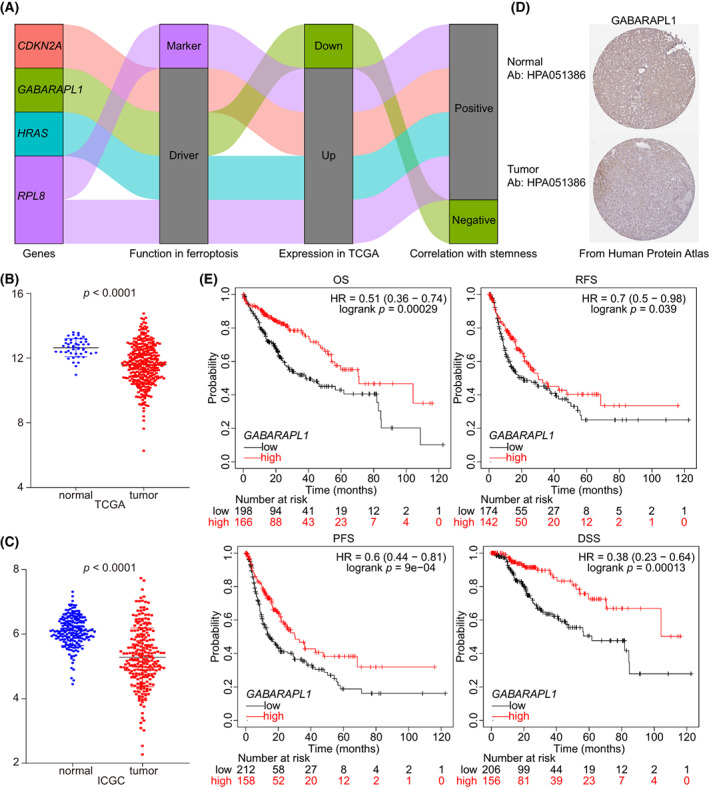
*GABARAPL1* was downregulated in HCC and had a negative correlation with prognosis. (A) Sankey diagram showing the role four hub genes in ferroptosis, expression in TCGA and their correlation with stemness. (B, C) Expression of *GABARAPL1* in TCGA (normal, *n* = 47, tumor, *n* = 345; B) and ICGC (normal, *n* = 205, tumor, *n* = 240; C) dataset (Mann–Whitney test). The error bars indicate SD. (D) Data from the HPA database showed the expression of *GABARAPL1* protein in HCC (*n* = 1): Normal tissue (https://www.proteinatlas.org/ENSG00000139112‐GABARAPL1/tissue/liver#img); tumor tissue (https://www.proteinatlas.org/ENSG00000139112‐GABARAPL1/pathology/liver+cancer#img). (E) Survival analysis of *GABARAPL1* in HCC. HCC, hepatocellular carcinoma; HPA, human protein atlas; ICGC, International Cancer Genome Consortium; TCGA, The Cancer Genome Atlas.

Ferroptosis resistance was considered characteristic of CSLC [6]. In line with this, we selected *GABARAPL1* as the focus of our further study, due to three features: driver factor of ferroptosis; downregulation in HCC; negative correlation with stemness (Fig. 5A). In detail, *GABARAPL1* was downregulated in HCC in both mRNA and protein level (Fig. 5B–D). Data from the Kaplan–Meier Plotter online tool showed that patients with high *GABARAPL1* expression had longer OS (71.0 *vs*. 38.3 months, *P* = 0.00029), recurrence‐free survival (RFS, 30.4 *vs*. 21.2 months, *P* = 0.039), progression free survival (PFS, 30.4 *vs*. 14.3 months, *P* < 0.0001) and disease‐free survival (DSS, 59.7 *vs*. 22.0 months, *P* = 0.00013) than those with low *GABARAPL1* expression (Fig. 5E). Setting tumor stage as a stratified factor, the expression of *GABARAPL1* was also significant correlated with HCC OS, RFS, PFS, and DSS (Suporting Information Fig. S1A,B). Hepatitis B virus (HBV) infection and alcohol abuse are the major causes of HCC. The expression of *GABARAPL1* also had a clinically and statistically significant prognostic value in all subgroups when taking alcohol consumption as a stratified factor (Fig. S1C). Of note, setting HBV infection as a stratified factor, *GABARAPL1* only achieved a significantly prognostic value in patients without HBV infection, but not in patients with HBV infection (Fig. S1D). We also analyzed the correlation between *GABARAPL1* expression and immune infiltration in HCC. Results showed that the expression of *GABARAPL1* had a negative correlation with most immune cells (e.g. B cells, T cells and macrophages) but a positive correlation with stromal cells (e.g. fibroblasts and endothelial cells, Supporting Information Fig. S2A,B). Consequently, low *GABARAPL1* expression was considered an indicator of poor prognosis in HCC.

### 3.5 HCC cells with high *GABARAPL1* expression was more vulnerable to ferroptosis

**Fig. 6:**
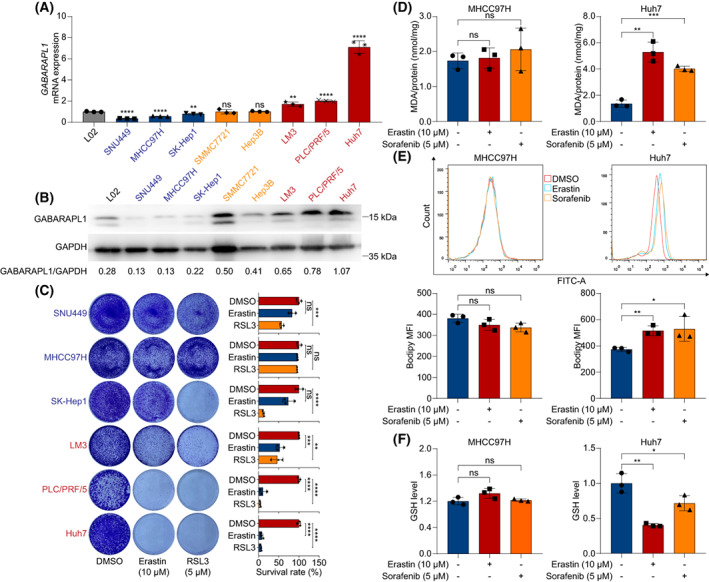
The expression of predicted *GABARAPL1* sensitivity to ferroptosis in HCC. (A) The mRNA level of *GABARAPL1* was determined using qRT‐PCR. (B) The protein level of *GABARAPL1* was detected using western blotting. (C) Colony formation assay assessed the sensitivity of HCC cell lines to erastin and RSL3. (D) Indicated cells were treated with erastin or sorafenib for 24 h and MDA was determined. (E) Lipid ROS level of indicated HCC cells treated with erastin or sorafenib for 24 h. (F) Relative GSH level in indicated cells treated with erastin or sorafenib for 24 h. Data are shown as mean ± SD. All experiments were repeated three times. The *t*‐test was used to determine the differences between two groups. HCC, hepatocellular carcinoma; ROS, reactive oxygen species; GSH, glutathione. *P < 0.05, **P < 0.01, ***P < 0.001, ****P < 0.0001.

To explore its function further, the expression of *GABARAPL1* was determined in HCC cell lines. *GABARAPL1* mRNA was significantly downregulated in SNU449, MHCC97H and SK‐Hep1 cell lines, and significantly upregulated in LM3, PLC/PRF/5 and Huh7 cell lines, but was not significantly changed in SMMC7721 and Hep3B when compared with normal human liver cell line L02 (Fig. 6A). Western blot showed similar alterations of *GABARAPL1* proteins in HCC cell lines (Fig. 6B). Colony formation assay demonstrated that erastin effected a moderate growth suppression of HCC cell lines with low *GABARAPL1*, and strong inhibited HCC cell lines with high *GABARAPL1* (Fig. 6C). MHCC97H (low *GABARAPL1*) and Huh7 (high *GABARAPL1*) were selected to determine the correlation between *GABARAPL1* expression and lipid peroxidation. Data from MDA assay and flow cytometry showed that *GABARAPL1* may enhance erastin‐induced lipid peroxidation (Fig. 6D,E). In addition, erastin triggered a more obvious decrease of GSH level in Huh7 cells than in MHCC97H cells (Fig. 6F). We next overexpressed *GABARAPL1* in MHCC97H cells, and silenced *GABARAPL1* in Huh7 cells (Fig. S3A,B). As shown, erastin did not have a significant influence on lipid peroxidation in *GABARAPL1*‐silenced Huh7 cells, but did trigger a significant alternation in *GABARAPL1*‐overexpressed MHCC97H cells (Fig. S3C–F). Collectively, *GABARAPL1* may sensitize HCC cells to erastin.

### 3.6 TRC‐acquired ferroptosis resistance due to downregulated *GABARAPL1*

**Fig. 7:**
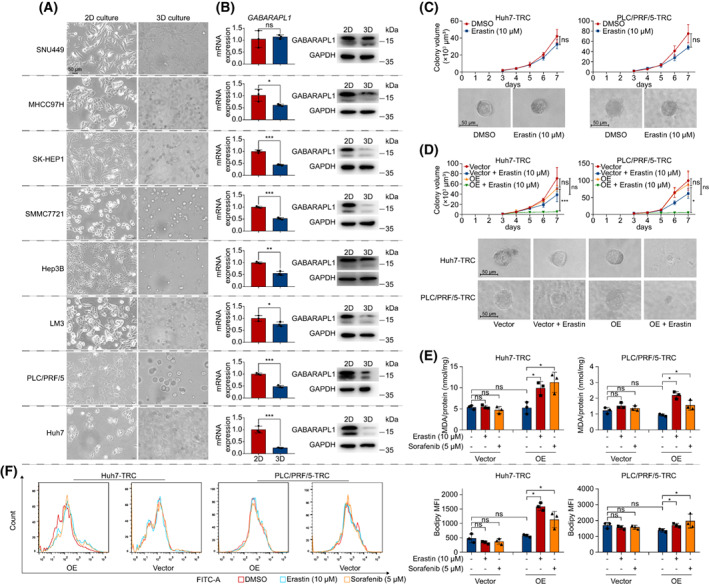
Downregulation of *GABARAPL1* in HCC‐TRC involved in ferroptosis resistance. (A) Cell morphology of 2D and 3D cultured HCC cells. (B) The mRNA and protein level of *GABARAPL1* in 2D cultured HCC cell and HCC TRC. (C) 2D cultured HCC cells were seeded in soft 3D fibrin gels to obtain TRC. On day 3, TRC were treated with erastin and then the colony spheroid was recorded on the next 4 days (days 3–7). The representative images of HCC‐TRC colony spheroids are from day 7. (D) Overexpression of *GABARAPL1*‐enhanced TRC sensitivity to erastin. (E, F) 2D cells were seeded in soft 3D fibrin gels and received lentivirus‐mediated transfection simultaneously. After 3 days, TRC were treated with erastin or sorafenib for 24 h. and the MDA (E) and lipid ROS (F) levels detected. Except western blot (*n* = 1), other experiments were independently repeated three times. Data were shown as mean ± SD. Statistics were performed by *t*‐test. Scale bar: 50 μm. HCC, hepatocellular carcinoma; ROS, reactive oxygen species; TRC, tumor‐repopulating cells. *P < 0.05, **P < 0.01, ***P < 0.001.

We investigated the correlation between *GABARAPL1* and stemness in HCC. We found that, with the exception of SNU449 cells, the remaining seven HCC cells emerged a typical spheroidizing growth in 3D soft fibrin gels (Fig. 7A). Consistently, compared with 2D cultured cells, the lower expression of *GABARAPL1* mRNA and proteins in TRC was observed in seven HCC cell lines, but not in SNU449 cells (Fig. 7B). Considering the sensitivity to erastin, Huh7 and PLC/PRF/5 cells were selected for further estimations. As anticipated, erastin barely suppressed the colony spheroids of Huh7‐TRC and PLC/PRF/5‐TRC (Fig. 7C). To confirm the underlying function of *GABARAPL1*, lentivirus‐mediated transfection was applied to transfer *GABARAPL1* plasmid into TRC, which was demonstrated by western blot and qRT‐PCR (Fig. S4). We found that overregulation of *GABARAPL1* promoted sensitivity of Huh7‐TRC and PLC/PRF/5‐TRC to erastin treatment (Fig. 7D). Under erastin treatment, more lipid peroxidation occurred in *GABARAPL1*‐overexpressed Huh7‐TRC and PLC/PRF/5‐TRC (Fig. 7E,F) than in the empty vector group. These results indicated that downregulating *GABARAPL1* confers ferroptosis resistance to HCC‐TRC, and upregulating it will recover sensitivity.

### 3.7 *Gabarapl1* expression was concerned with sorafenib sensitivity

**Fig. 8:**
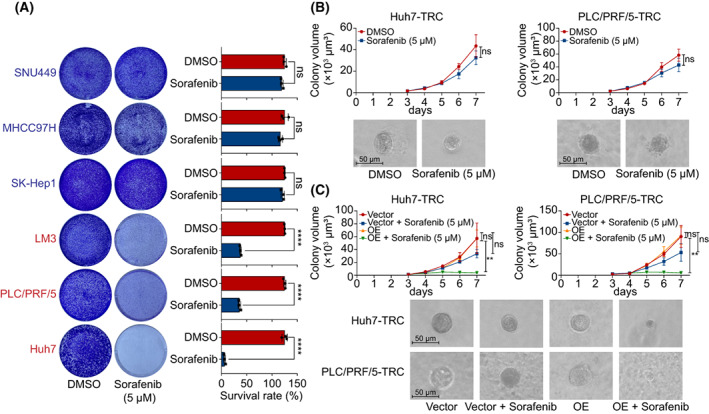
The association of *GABARAPL1* with sorafenib sensitivity. (A) Colony formation assay assessed the sensitivity of HCC cell lines to sorafenib. (B) Sorafenib barely inhibited colony spheroids formation in Huh7‐TRC and PLC/PRF/5‐TRC. (C) Overexpression of *GABARAPL1* boosted sorafenib inhibition activity against colony spheroid formation in HCC‐TRC. All experiments were independently repeated three times. Data are shown as mean ± SD and statistics were performed by *t*‐test. Scale bar: 50 μm. HCC, hepatocellular carcinoma; TRC, tumor‐repopulating cells; **P < 0.01, ****P < 0.0001.

The above results indicated the correlation between *GABARAPL1* and ferroptosis sensitivity. Of note, the expression of *GABARAPL1* only predicted HCC sensitivity to erastin‐induced ferroptosis, not to RSL3‐induced ferroptosis (Fig. 6C). Sorafenib shared a similar action mode to erastin, i.e. inhibition of system x_c_^−^ [21]. Hence, we hypothesized that *GABARAPL1* also regulates the sensitivity of HCC cells to sorafenib. To this end, we investigated the efficacy of sorafenib on 2D cultured HCC cells and found that low *GABARAPL1* cells were resistant to sorafenib, whereas high *GABARAPL1* cells were sensitive to sorafenib, consistent with erastin treatment (Fig. 8A). MDA assay and flow cytometry revealed that sorafenib induced more lipid peroxidation in Huh7 cells than in MHCC97H cells, indicating more ferroptosis occurred in high‐expressing HCC cells (Fig. 6D,E). Sorafenib also resulted in more reduction of GSH in high‐expressing HCC cells (Fig. 6F). Moreover, overexpressing *GABARAPL1* improved the sensitivity of MHCC97H to sorafenib, whereas silencing *GABARAPL1* decreased the sensitivity of Huh7 to sorafenib (Fig. S2C–F). Accordingly, sorafenib‐induced ferroptosis was associated with the expression of *GABARAPL1*.

We and others suggested that CSLC made a large contribution to sorafenib resistance in HCC (Fig. 8B) [22]. Of importance, enforced overexpression of *GABARAPL1* restored the sensitivity of Huh7‐TRC and PLC/PRF/5‐TRC to sorafenib treatment (Fig. 8C). More specifically, sorafenib treatment elicited more lipid peroxidation in *GABARAPL1*‐overexpressed Huh7‐TRC, as well as PLC/PRF/5‐TRC (Fig. 7E,F).

## 4 Discussion

We investigated a series of FRSG and divided HCC samples in two clusters based on these FRSG. C1 type HCC possessed low immune infiltration and ICI response rate but high TMB, and led to a poorer prognosis compared with C2 type HCC. We further constructed a four‐gene RS model (*CDKN2A*, *GABARAPL1*, *HRAS*, *RPL8*) which gave a good prediction of HCC patient outcome. Among these genes, we found that *GABARAPL1* was correlated with HCC cells sensitivity to ferroptosis inducers. Most importantly, *GABARAPL1* was downregulated in HCC TRC that were resistant to ferroptosis inducers, and overexpression of the gene recovered ferroptosis sensitivity in these cells. These data suggest that loss of *GABARAPL1* may contribute to ferroptosis resistance in HCC CSLC.

In the past year, dozens of bioinformatics articles investigated the correlation of FRG in cancers 23, 24, 25] For instance, Liang et al. constructed a 10‐FRG RS model that effectively predicted HCC patient prognosis in both discovery and validation cohorts, and showed that high‐risk patients had attenuated antitumor immunity [23]. In this article, the difference was that we focused on FRSG, a set of genes related to ferroptosis as well as stemness. According to these FRSG, HCC samples were classified into two clusters, C1 and C2. Tumor microenvironment analysis indicated that a higher immune infiltration was observed in C2 than in C1 type HCC. C2 type HCC had a higher level of most immune checkpoints, which may explain its high response to ICI predicted by TIDE. In addition, C2 type had longer OS and lower TMB, consistent with a previous study showing a negative correlation of TMB with HCC prognosis [26]. These data demonstrated that FRSG also provided a well typing scheme in HCC.

We also established a four‐FRSG RS model. This model displayed an excellent prediction of HCC patient prognosis in discovery and external validation cohorts. According to the FerrDb dataset, the four FRSG may have a role as a ferroptosis driver in cancer [9]. Differential gene analysis and OCLR algorithm revealed that *CDKN2A*, *HRAS* and *RPL8* were upregulated in HCC and that there was a positive association with stemness. However, *GABARAPL1* was downregulated in HCC and had a negative association with stemness. As noted, CSLC may confer resistant features to ferroptosis [6]. In addition, we and others have found that *GABARAPL1* was negatively related to HCC patient prognosis [27]. For these reasons, we selected *GABARAPL1* as the focus of our further experiments. *GABARAPL1* was a member of ATG8 (autophagy‐related 8)‐family that was described to be involved in autophagosome assembly, elongation, membrane curvature, autophagosome closure, the fusion between autophagosome and lysosome, and for selective autophagy [28]. Previous study indicated that *GABARAPL1* acts as a tumor suppressor protein, and showed that it inhibited cell proliferation, invasion and tumor growth in breast cancer and prostate cancer [28, 29, 30]. However, its function in HCC remains unclear. Here, we found that the expression of *GABARAPL1* affected the sensitivity of HCC cells to the ferroptosis‐inducer erastin. More importantly, HCC TRC had a lower *GABARAPL1* expression than its parent 2D cultured cells, and displayed ferroptosis resistance under erastin treatment. Recovering its expression enhanced TRC sensitivity to erastin‐induced ferroptosis. These data indicated that loss of *GABARAPL1* may confer ferroptosis resistance to CSLC in HCC. Convincing evidence revealed that autophagy, especially a selective type of autophagy (e.g. ferritinophagy, lipophagy, clockophagy and chaperone‐mediated autophagy), drives cells towards ferroptosis by promoting iron accumulation or/and lipid peroxidation [31, 32, 33]. For instance, RAB7A‐dependent lipophagy boosts lipid droplet degradation and thus induces lipid peroxidation‐mediated ferroptosis [34]. Song et al. revealed that autophagy‐related protein BECN1 formed a complex with SCL7A11 and thereby inhibited system x_c_^−^ activity, contributing to cell death in response to erastin but not RSL3 [33]. Our data showed that the expression of *GABARAPL1* also cannot predict cell sensitivity to RSL3. In view of the known function of *GABARAPL1*, it may work by regulating system x_c_^−^ activity or/and autophagy (data not shown).

Sorafenib, a crucial clinical drug for HCC, was considered as a potential ferroptosis inducer achieved through inhibiting system x_c_^−^ activity [21]. Resistance to sorafenib seriously limits the benefits of HCC patients. The mechanisms of sorafenib resistance are various; cancer stem cells or CSLC may be one of the important factors [22, 35, 36]. We demonstrated that sorafenib only produced a slight inhibition against spheroidizing growth in HCC TRC. Overexpression of *GABARAPL1* significantly enhanced sorafenib inhibition activity against HCC TRC due to the increased ferroptosis. These data suggested that acquired ferroptosis resistance may be the explanation for sorafenib resistance in HCC CSLC. Further research into *GABARAPL1* may provide novel strategies for overcoming sorafenib resistance in HCC.

Several limitations of the study should be noted. First, *GABARAPL1* is a well‐known autophagy‐related gene, and autophagy can drive cell ferroptosis. It is unclear whether autophagy plays a role in *GABARAPL1*‐related ferroptosis. Secondly, although *in vitro* data indicated that increasing *GABARAPL1* expression effectively sensitized HCC CSLC to erastin or sorafenib, *in vivo* testing will provide more solid evidence.

## 5 Conclusions

In this paper, we put forward a new classification scheme for HCC based on FRSG and dividing HCC samples into two clusters. The two clusters had different responses to ICI, which may contribute to guiding immunotherapy in HCC. In addition, a four‐FRSG RS model with a significant prognosis signature was constructed in HCC. Finally, we mined an interesting FRSG *GABARAPL1* and found that its downregulation conferred ferroptosis resistance to HCC CSLC under erastin or sorafenib treatment. Further study of this gene may be conducive to developing a potential strategy to overcome sorafenib resistance in HCC.

## Conflict of interest

The authors declare no conflict of interest.

## Author contributions

XD, ZQ, JX (Jinzhi Xu) and XZ contributed to the experiments. XD and MG were responsible for bioinformatics analysis. XD contributed to writing the article. XC and JX (Jinglin Xia) designed the project ideas, provided technical guidance and guided figure layout. ZY contributed to the additional experiments and paper revision.

### Peer Review

The peer review history for this article is available at https://publons.com/publon/10.1002/1878‐0261.13305.

## Supporting information


**Fig. S1.** Stratified survival analysis of *GABARAPL1* in HCC.
**Fig. S2.** Correlation between immune infiltration and *GABARAPL1* in HCC.
**Fig. S3.** Interference with expression of *GABARAPL1* affected the sensitivity to ferroptosis in HCC.
**Fig. S4.** The overexpression efficiency in TRC.

## Data Availability

RNA‐seq data were downloaded from TCGA (https://portal.gdc.cancer.gov/, TCGA‐LIHC) and ICGC (https://dcc.icgc.org/projects/LIRI‐JP) database. FRG were obtained from FerrDb database (http://www.zhounan.org/ferrdb/legacy/index.html#). The protein expression of *GABARAPL1* in normal tissue (https://www.proteinatlas.org/ENSG00000139112‐GABARAPL1/tissue/liver#img) and tumor tissue (https://www.proteinatlas.org/ENSG00000139112‐GABARAPL1/pathology/liver+cancer#img) was from HPA database (https://www.proteinatlas.org/). Survival analysis of *GABARAPL1* was from the Kaplan–Meier Plotter online tool (http://kmplot.com/analysis/index.php?p=background).
