# Supplementary material for: Loss of GABARAPL1 confers ferroptosis resistance to cancer stem‐like cells in hepatocellular carcinoma
Source: Mol Oncol. 2022 Sep 5;16(20):3703–19. doi: 10.1002/1878-0261.13305 (PMC9580891; doi:10.1002/1878-0261.13305)
Supplement: Supplementary file 1 — Fig. S1. Stratified survival analysis of GABARAPL1 in HCC. Fig. S2. Correlation between immune infiltration and GABARAPL1 in HCC. Fig. S3. Interference with expression of GABARAPL1 affected the sensitivity to ferroptosis in HCC. Fig. S4. The overexpression efficiency in TRC. [file MOL2-16-3703-s001.docx]

**Supplemental figure legends**

**Supplemental figure 1. Stratified survival analysis of GABARAPL1 in HCC.** A–D. Taken tumor stage (A & B), alcohol consumption (C) and HBV infection (D) as stratified factors, the prognosis value of *GABARAPL1* was estimated in HCC. HCC, hepatocellular carcinoma; HBV, Hepatitis B virus.

**Supplemental figure 2. The correlation between immune infiltration and GABARAPL1 in HCC.** A. The lollipop graph showed the results of Pearson correlation analysis between immune infiltration and *GABARAPL1* expression in HCC. B. The heatmap showed the trend of immune infiltration with the increased expression level of *GABARAPL1*.

**Supplemental figure 3. Interfering the expression of GABARAPL1 affected the sensitivity to ferroptosis in HCC.** A & B. The expression interference efficiency against *GABARAPL1* was detected by using qRT-PCR and western blotting. C & D. *GABARAPL1*-silencing Huh7 (C) and *GABARAPL1*-overexpressing MHCC97H (D) cells were treated with erastin or sorafenib for 24 hours and MDA was detected. E & F. *GABARAPL1*-silencing Huh7 (E) and *GABARAPL1*-overexpressing MHCC97H (F) cells were treated with erastin or sorafenib for 24 hours. Lipid ROS level was detected by using flow cytometry. The statistical analysis was performed by using t test. HCC, hepatocellular carcinoma; ROS, reactive oxygen species. *p < 0.05, **p < 0.01, ***p < 0.001.

**Supplemental figure 4. The overexpressing efficiency in TRCs.** A–D. Huh7-TRC and PLC/PRF/5-TRC were transfected with lentivirus-based *GABARAPL1* plasmid. The efficiency was detected by using qRT-PCR and western blotting. The statistical analysis was performed by using t test. TRCs, tumor-repopulating cells. ****p < 0.0001.

**Supplemental figure 1**


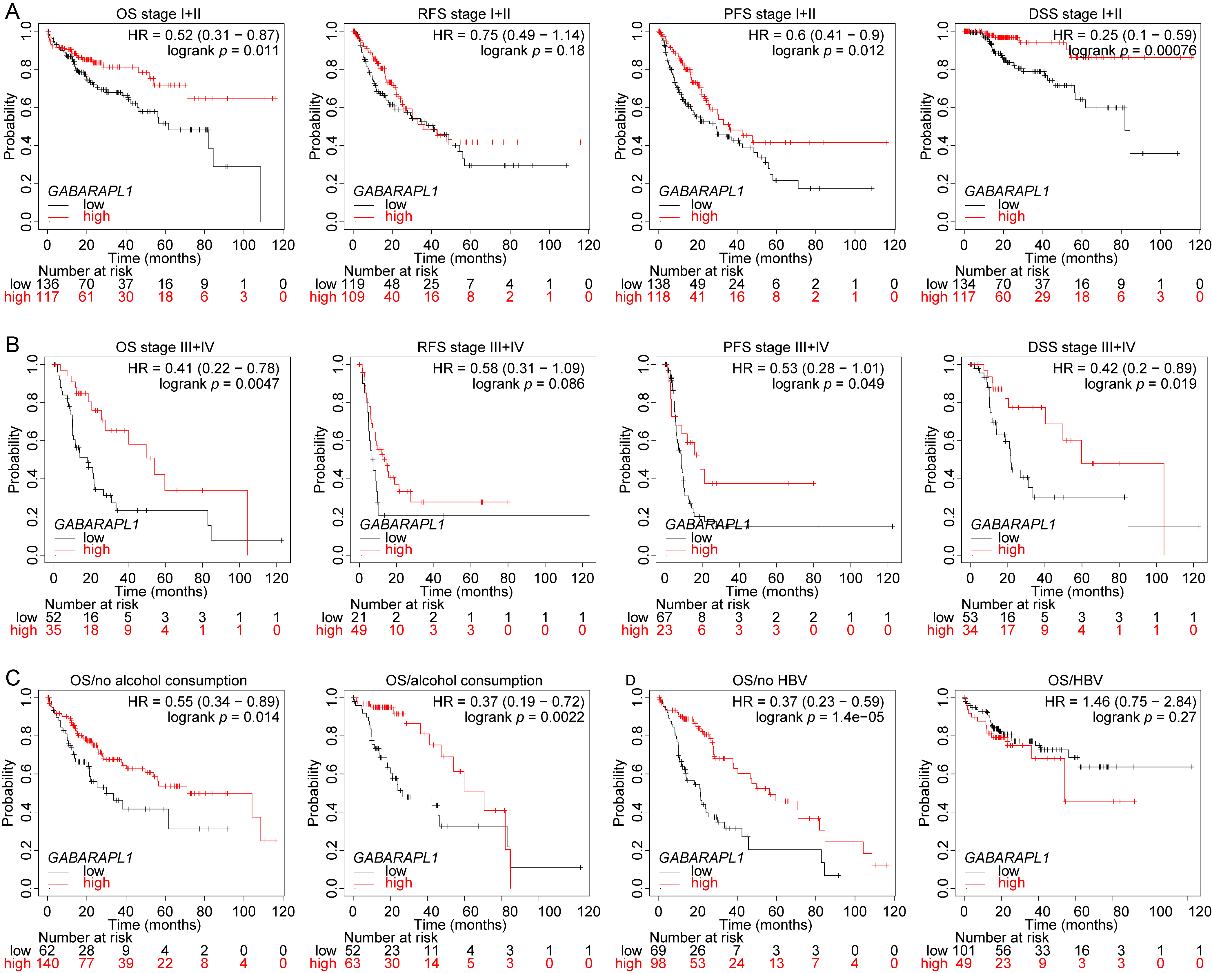


**Supplemental figure 2**


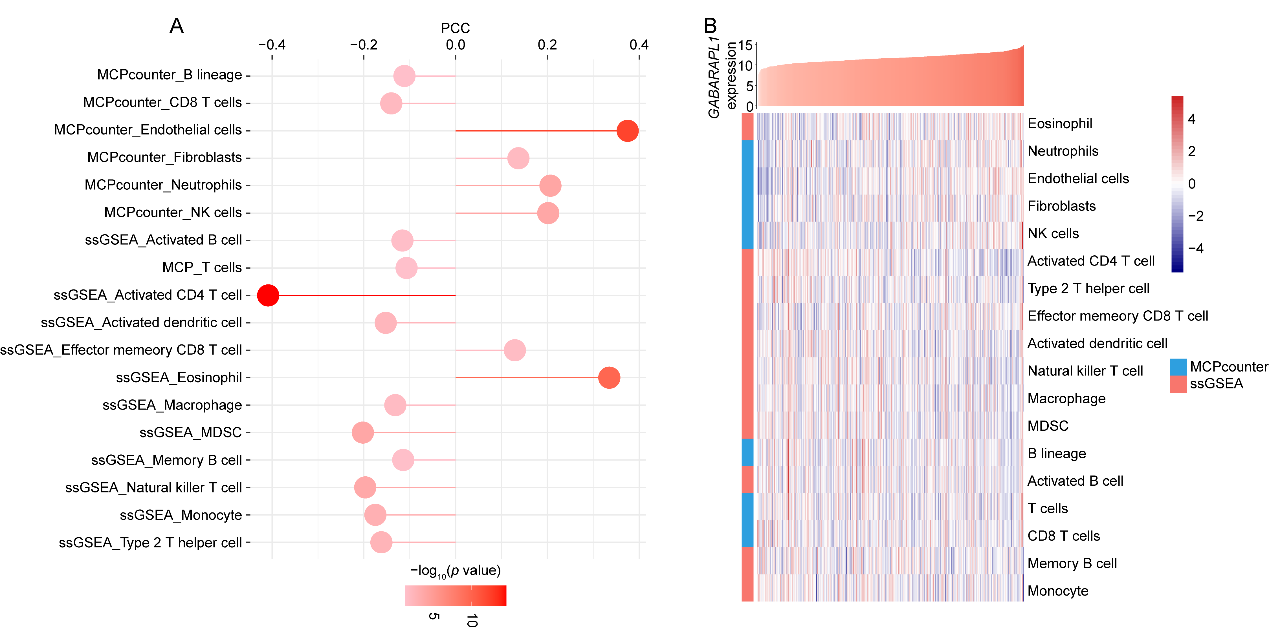


**Supplemental figure 3**

**
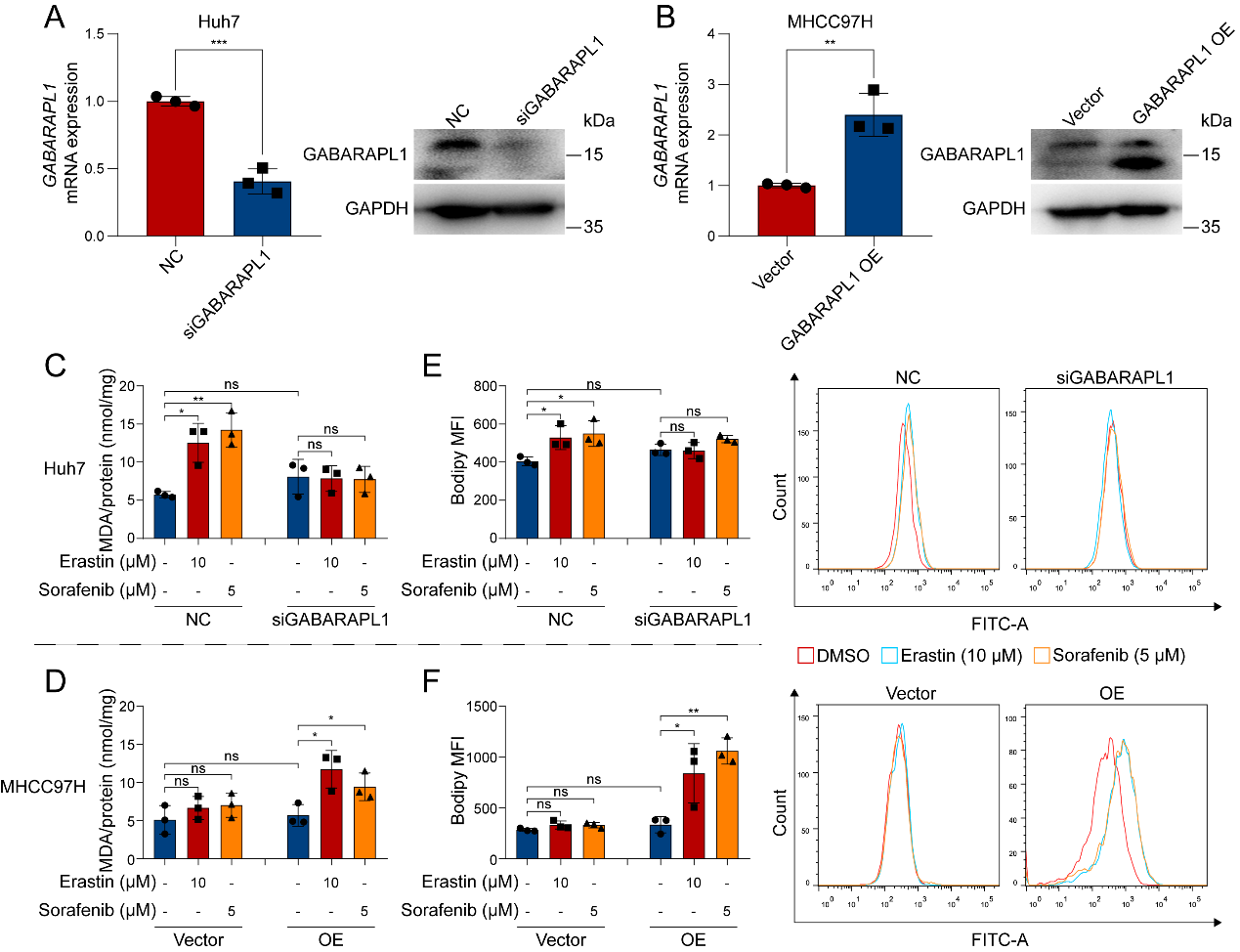
**

**Supplemental figure 4**

**
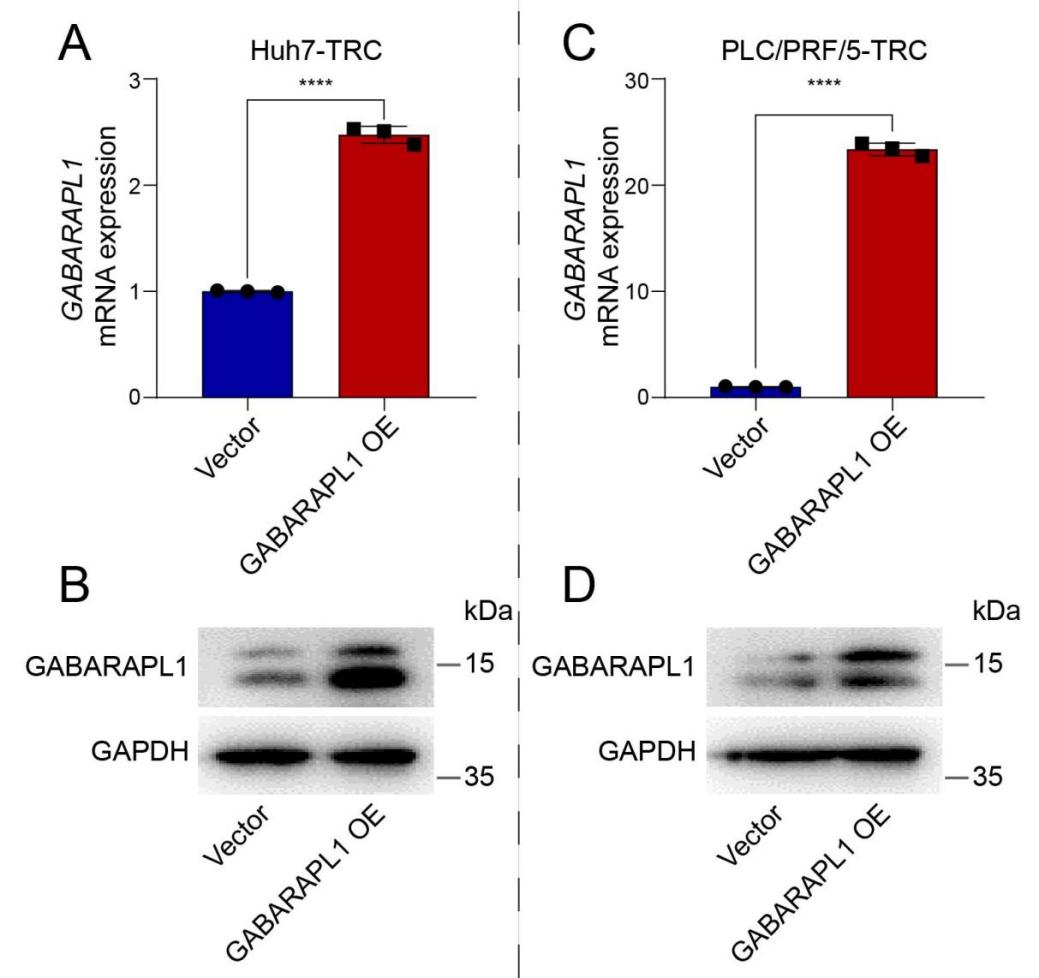
**
